# Scapular Fractures at a Level 1 Trauma Center: A Cross-Sectional Study

**DOI:** 10.5339/qmj.2022.8

**Published:** 2022-03-02

**Authors:** Hammam Kayali, Abdulaziz F. Ahmed, Talal Ibrahim

**Affiliations:** ^1^Section of Orthopaedics, Department of Surgery, Hamad General Hospital, Doha, Qatar E-mail: tibrahim@sidra.org; ^2^Division of Orthopaedic Surgery, Department of Surgery, Sidra Medicine, Doha, Qatar

**Keywords:** Scapula,, fractures, cross-sectional, operative, level 1, trauma, center

## Abstract

Purpose: Scapular fractures are uncommon injuries that account for up to 1% of all fractures and 5% of all shoulder girdle fractures. Moreover, most of the evidence on the treatment of scapular fractures stems from case series, with paucity of comparative studies. Despite the lack of standardized criteria for the operative treatment of scapular fractures, a set of suggested radiological parameters has been recently reported. The primary aim of this study was to compare the treatment implemented for scapular fractures in comparison with standard published criteria. The secondary aim was to investigate epidemiological parameters of scapular fractures at a level 1 trauma center.

Methods: In this cross-sectional study of scapular fractures at a level 1 trauma center, data were collected between December 2012 and January 2016. Data of all scapular fractures that presented to our center were retrospectively collected through electronic medical records. Identified cases of scapular fractures were then evaluated whether surgical treatment was indicated in accordance with recent standard operative criteria. Percentages were used to express the number of cases that were operatively indicated according to the predefined criteria and the number of cases operatively treated at our institution.

Results: A total of 52 patients met the inclusion criteria of having scapular fractures documented on radiography and Computed tomography (CT). The mean age of the patients was 38.5 years, with the majority being men (92.3%). The most common mechanism of injury was a fall from a considerable height in 26% of the cases. Of the included patients, 53.8% were polytraumatized, and the most frequent concomitant traumatic injury was rib fractures (26.9%). Only 33% of intra-articular glenoid fractures with significant displacement were treated operatively. Furthermore, non-operative treatment was undertaken in indicated extra-articular scapular body and neck fractures, acromion or coracoid process fractures, or superior shoulder suspensory complex double disruptions.

Conclusion: A significant discrepancy was found between the treatments implemented at our institution and the current standard criteria for the operative treatment of scapular fractures. This study emphasizes the need to educate surgeons on scapular fractures and to treat such fractures in accordance with standard published criteria. Furthermore, scapular fractures that require surgery should be referred to a surgeon experienced in scapular fracture fixation.

## Introduction

Scapular fractures are uncommon injuries that account for up to 1% of all fractures and up to 5% of all shoulder girdle fractures.^1^ Scapular fractures are most prevalent in young men (aged 20–56.6 years).^2–4^ High-energy trauma is implicated in most scapular fractures following motor vehicle accidents and falls (80%–88%).^5,6^ Given the involvement of high-energy trauma, scapular fractures are often associated with concomitant injuries, such as rib fractures, upper limb injuries, spine injuries, pneumothorax, pulmonary contusion, and neurovascular compromise.^1,7^

Most scapular fractures affect the scapular body (45%), followed by the glenoid (35%), and the least affected parts are the acromion and coracoid.^8^ Scapular fractures are initially evaluated with shoulder radiography. Computed tomography (CT) of the scapula is required to further evaluate fracture patterns, particularly fractures with an intra-articular glenoid component. Various anatomical classification systems have been introduced to guide the treatment of scapular fractures. The Orthopedic Trauma Association classification is a comprehensive system of all scapular fractures and is often used for research purposes.^9^ Other classifications focus on a specific part of scapular fractures, such the Ideberg classification that concentrates on intra-articular glenoid fractures.^2^

The most displaced extra-articular fractures are often treated non-operatively. However, over the past two decades, there has been a shift toward surgical treatment of such fractures owing to the better understanding of scapular kinematics and improvement in both surgical techniques and implants.^10^ Most of the evidence on the treatment of scapular fractures stems from case series, with paucity of comparative studies.^11,12^ Despite the lack of standardized criteria for the operative treatment of scapular fractures, a set of suggested radiological parameters have been recently reported.

The primary aim of this study was to compare the treatment implemented for scapular fractures in comparison with standard published criteria. The secondary aim was to investigate epidemiological parameters of scapular fractures at our level 1 trauma center.

## Materials And Methods

### Study Design and Participants

This was a cross-sectional study of scapular fractures at Hamad General Hospital, Doha, Qatar, which is a level 1 trauma center. Data were collected between December 2012 and January 2016. Data of all scapular fractures that presented to our center were collected retrospectively through electronic medical records. The inclusion criteria were age ≥ 18 years and scapular fractures documented on shoulder anteroposterior (AP) and scapula Y radiography and scapular CT. The exclusion criterion was the lack of adequate radiographic views or CT scans.

### Variables

Variables such as age, sex, mechanism of injury, and fractured portion of the scapula (glenoid, body, coracoid, or acromion) were recorded. To determine whether a fracture met the operative indications, radiographic parameters were measured. Glenoid intra-articular step-off and the involved portion of the glenoid surface were measured on CT scans of the scapula. The intra-articular step-off measures the amount of displacement of the fractured portion of the intra-articular glenoid. Radiographs were used to measure scapular body medialization, scapular body angulation in the semicoronal plane, glenopolar angle, and presence of superior shoulder suspensory complex (SSSC) disruption. On AP shoulder radiographs, scapular body medialization was measured as the amount of medial displacement of the glenoid segment relative to the midline of the body. The glenopolar angle was measured on AP shoulder radiographs as the angle between a line parallel to the glenoid surface and a line connecting the glenoid with the inferior angle of the scapula. Acromion fractures, coracoid fractures, and double disruptions of the SSSC were evaluated by reviewing displacement on both shoulder radiographs and CT scans.

### Statistical Analysis

Baseline characteristics were expressed with summary statistics such as means with standard deviations for continuous variables, whereas percentages were used for categorical variables.

To achieve the primary outcome, identified cases of scapular fractures were evaluated to determine whether surgical treatment was indicated in accordance with the recent standard operative criteria.^13^ The suggested recent operative criteria were as follows: 1) intra-articular glenoid fractures with a step-off >4 mm and involvement of 25% of the glenoid surface, 2) scapular body fractures with medialization ≥  20 mm, 3) scapular body fractures with semicoronal plane angulations of 45°, 4) combined medialization of 15 mm and semicoronal plane angulation of 30°, 5) glenopolar angle < 22°, and 5) double disruptions of SSSC with 10-mm displacement for both disruptions. Thereafter, percentages were used to express the number of cases that were operatively indicated according to the predefined criteria and the number of cases operatively treated at our institution.

## Results

**Figure 1.**
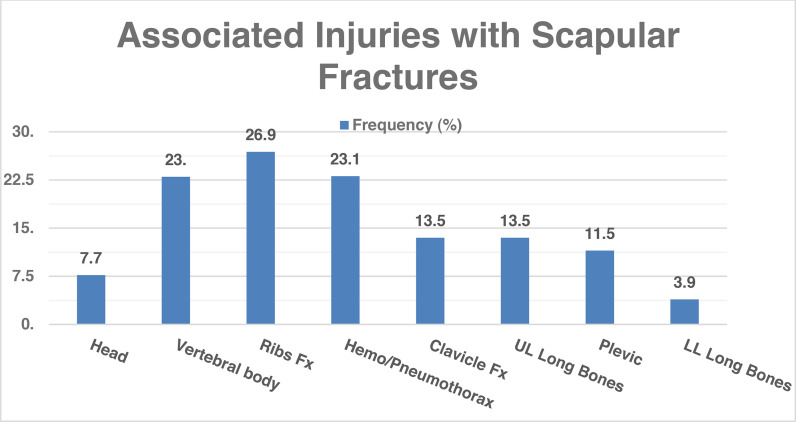
Concomitant injuries with scapular fractures. Fx, fracture; UL, upper limb; LL, lower limb.

A total of 52 patients met the inclusion criteria, i.e., scapular fractures documented by radiographs and CT scans. At the time of injury, the mean age of the patients was 38.5 years. The mean age was 37.3 years +/ −  11.1 in male patients and 52.5 years +/ −  22.8 in female patients. Most patients were men (92.3%; N = 48). The most common mechanism of injury was a fall from a considerable height in 26%, followed by motor vehicle accidents in 25%, and a slip and fall in 15% of the cases. Of the 52 patients, 26 (53.8%) had multiple traumatic injuries. The most frequent concomitant traumatic injuries were rib fractures (26.9%), followed by hemothorax and/or pneumothorax (23.1%) and spine vertebral body fractures (19.2%) (Figure 1).

**Figure 2.**
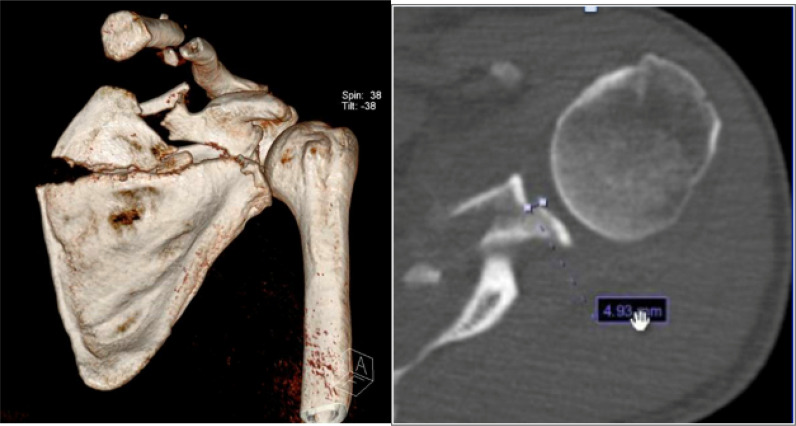
Three-dimensional reconstruction and axial computed tomography scan of a patient with left glenoid displaced fracture that was treated nonoperatively.

**Figure 3.**
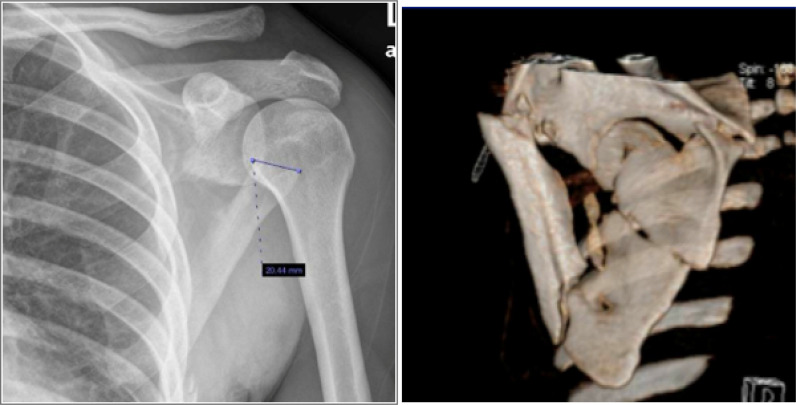
Anteroposterior shoulder radiograph and three-dimensional reconstruction image of a patient with extra-articular fracture that was treated nonoperatively.

**Figure 4.**
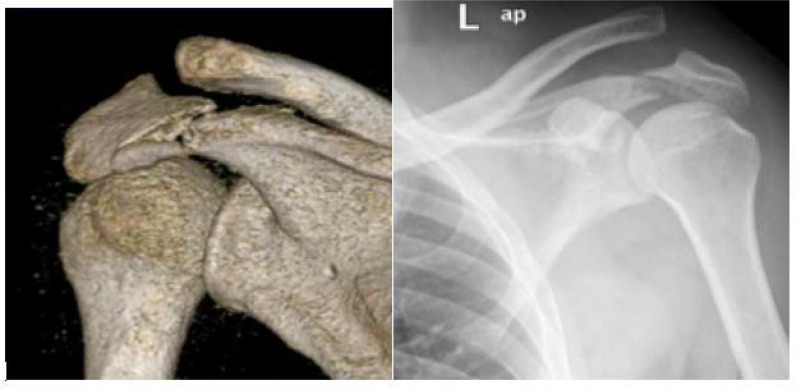
Three-dimensional reconstruction view and shoulder anteroposterior radiograph of a patient with left acromion-displaced fracture and acromioclavicular joint dislocation that was treated nonoperatively.

As regards to fracture types, intra-articular glenoid fractures were present in 18 cases (34.6%), extra-articular scapular body fractures in 27 (51.9%), process fractures in 25 (48.1%), and SSSC injuries in 4 (7.6%), indicating that a patient can have more than one type of scapular fracture concomitantly. For intra-articular glenoid fractures, 50% (N = 9) of the patients met the operative indications of a step-off >4 mm and/or glenoid involvement >25%. In this case series, only 33.3% (3 of 9 cases) of the patients with displaced intra-articular glenoid fractures were treated operatively (Figure 2). As regards to extra-articular scapular body and neck fractures, the operative criteria were scapular body fractures with medialization ≥ 20 mm, scapular body fractures with a semicoronal plane angulation of 45°, combined medialization of 15 mm and semicoronal plane angulation of 30°, or glenopolar angle < 22°. In this case series, only 33.3% (9 of 27) of the patients met the operative criteria of extra-articular scapular body fractures. However, no patients underwent operative treatment with such fractures in our study (Figure 3). For scapular process fractures and SSSC double disruption injuries, the operative criteria included any displacement >10 mm. Twenty-five patients had coracoid and acromion process fractures, of which only 16.6% (4 of 24) met the operative indications. Furthermore, double disruptions of the SSSC were observed in 4 patients, in which 1 (25%) of these patients met the operative criteria. In our series, no patient that had an indicated process fracture or SSSC double disruptions was treated operatively (Figure 4). Table 1 summarizes the total number of patients with each fracture pattern and the number of operatively indicated and treated cases.

## Discussion

This study revealed that operative treatment was implemented only in a minority of scapular fractures that met the operative indications. The only fracture types that were addressed were displaced glenoid intra-articular fractures; however, only 33% of the indicated intra-articular fractures were treated operatively. None of the indicated scapular body extra-articular fractures, coracoid fractures, acromion fractures, or SSSC double disruptions were managed operatively. The undertreatment of the indicated scapular fractures at our institution is attributable to several reasons. It is well recognized that nearly 90% of all scapular fractures are minimally displaced.^14^ The scarcity of operatively indicated scapular fractures would pose a significant challenge to unfamiliar orthopedic surgeons. Moreover, the wide range of motion of the shoulder joint would compensate if a scapular malunion or nonunion would occur. The paucity of knowledge of the scapula's complex anatomy and kinematics and inexperience with surgical approaches to the scapula promotes non-operative treatment and benign neglect of such fractures. The evidence on the treatment of scapular fracture remains contradicting with several reports supporting both operative and non-operative treatments.^11^ The current evidence stems from case series without randomized trials and paucity of comparative cohorts.

In our center, operative treatment was only implemented in a small proportion of glenoid intra-articular fractures among all scapular fractures. This is potentially because the literature is more comprehensible in the treatment of displaced intra-articular glenoid fractures and intra-articular fractures in general. A major concern of the non-operative treatment of such fractures is the risk of post-traumatic instability and osteoarthritis. However, in earlier reports, open reduction and internal fixation (ORIF) resulted in satisfactory outcomes for glenoid intra-articular fractures that were displaced by 2–5 mm.^15,16^ In a recent case series of 33 glenoid fractures with an intra-articular component with 5-mm displacement, Anavian et al. reported that operative treatment resulted in pain-free shoulders in 87% of the patients and return to the pre-injury level in 90% at the mean 27 months of follow-up.^17^

In comparison, the evidence becomes more controversial for extra-articular displaced scapular fractures. Such dispute in the literature is reflected in our series where operative treatment was not implemented in any of the displaced extra-articular fractures. In a comparative cohort by Jones and Sietsema, both open reduction internal fixation and non-operative treatments of displaced scapular neck and body fractures had similar outcomes.^18^ However, a major limitation in this study was the significantly higher initial displacement in the operative group. In a series of 18 scapular neck fractures, Bozkurt et al. reported that non-operative treatment had worse Constant scores and associated with lower glenopolar angle (r = 0.9; p < 0.05).^19^ When operative treatment was implemented for displaced extra-articular fractures, Herrera et al. found significant improvements in shoulder functional scores, range of motion, and strength at a follow-up of 24 months.^20^ In a more recent study, Schroder et al. achieved reliably satisfactory outcomes for severely displaced extra-articular scapular fractures that met published operative indications.^21^

This study has several limitations. First, the retrospective design might introduce misclassification bias in determining whether a fracture meets operative indications. Second, no shoulder functional outcomes were present, which precludes the comparison between operatively indicated fractures that were treated either operatively or non-operatively. Finally, the low proportion of scapular fractures that met surgical indications is not generalizable given that our results relied on the knowledge and technical expertise of the treating surgeons at our institution.

In conclusion, a significant discrepancy was found between treatments implemented at our institution and the currently standard criteria for scapular fractures that met operative indications. Only 33% of intra-articular glenoid fractures with significant displacement were treated operatively. Furthermore, non-operative treatment was undertaken in indicated extra-articular scapular body and neck fractures, acromion or coracoid process fractures, or SSSC double disruptions. Given the paucity of knowledge about the outcomes of undertreated scapular fractures, this study emphasizes the need to educate surgeons on scapular fractures and to treat scapular fractures in accordance with standard published criteria. Furthermore, scapular fractures that require surgery should be referred to a surgeon experienced in scapular fracture fixation. More studies are warranted to gain further understanding on the treatment outcomes of scapular fractures that meet the current standard criteria.

### Conflict of Interest

None

## Figures and Tables

**Table 1:** Summary of operatively indicated and treated patients.

Fracture	Total Number	Operatively indicated % (N)	Operatively treated % (N)

Intra-articular glenoid fractures	18	50% (9)	33.3% (3)

Extra-articular Scapular fractures	27	33.3 (9)	0%

Process fractures (Acromion or coracoid)	24	16.6 (4)	0%

SSSC double disruption	4	25.1 (1)	0%


